# Development of Automatic Visceral Fat Volume Calculation Software for CT Volume Data

**DOI:** 10.1155/2014/495084

**Published:** 2014-03-20

**Authors:** Mitsutaka Nemoto, Tusufuhan Yeernuer, Yoshitaka Masutani, Yukihiro Nomura, Shouhei Hanaoka, Soichiro Miki, Takeharu Yoshikawa, Naoto Hayashi, Kuni Ohtomo

**Affiliations:** ^1^Department of Radiology, The University of Tokyo Hospital, 7-3-1 Hongo, Bunkyo-ku, Tokyo 113-8655, Japan; ^2^Imaging Center, The Second Affiliated Hospital, Xinjiang Medical University, The 2Rd Xiang 38, Nan Hu Dong Road, Urumqi, Xinjiang 830063, China; ^3^Department of Computational Diagnostic Radiology and Preventive Medicine, The University of Tokyo Hospital, 7-3-1 Hongo, Bunkyo-ku, Tokyo 113-8655, Japan

## Abstract

*Objective*. To develop automatic visceral fat volume calculation software for computed tomography (CT) volume data and to evaluate its feasibility. *Methods*. A total of 24 sets of whole-body CT volume data and anthropometric measurements were obtained, with three sets for each of four BMI categories (under 20, 20 to 25, 25 to 30, and over 30) in both sexes. True visceral fat volumes were defined on the basis of manual segmentation of the whole-body CT volume data by an experienced radiologist. Software to automatically calculate visceral fat volumes was developed using a region segmentation technique based on morphological analysis with CT value threshold. Automatically calculated visceral fat volumes were evaluated in terms of the correlation coefficient with the true volumes and the error relative to the true volume. *Results*. Automatic visceral fat volume calculation results of all 24 data sets were obtained successfully and the average calculation time was 252.7 seconds/case. The correlation coefficients between the true visceral fat volume and the automatically calculated visceral fat volume were over 0.999. *Conclusions*. The newly developed software is feasible for calculating visceral fat volumes in a reasonable time and was proved to have high accuracy.

## 1. Introduction

The high correlation between the visceral fat volume and the development of arteriosclerotic diseases is well known [1–5]. Evaluation of the visceral fat volume is crucially important in research on arteriosclerotic diseases. Since direct measurement of the visceral fat volume is difficult at clinical sites, substitute indices such as the waist circumference (WC) at umbilicus level, the body mass index (BMI), and the area of the 2D visceral fat region on a CT/MR axial slice at the umbilicus level have been used to evaluate the visceral fat volume. However, the accuracy of these substitute indices is not yet well described in the medical literature. Wei et al. evaluated the three indices (WC, BMI, and percentage of body fat estimated by DXA method) with fasting serum insulin levels along with metabolic syndrome components [6]. Since the DXA method cannot separate visceral and subcutaneous fat, body fat is not only the visceral component but the total of the two components. On the other hand, Janssen et al. used MRI to measure the visceral fat volume and evaluate its association with WC and BMI [7]. Since they used a manual interactive method to segment the images, the whole visceral fat volume was estimated from only five images. An automatic method with a larger number of thin images would be necessary to calculate the volumes more accurately.

In recent years, multislice CT scanners have come into global use. To meet the growing demand for reconstructed images, CT volume data has been acquired and accumulated in many institutions. Using the accumulated CT volume data, it is possible to calculate a patient's visceral fat volume by manual segmentation of the visceral fat region in every slice of the CT volume data. However, since this manual calculation of the visceral fat volume is extremely time-consuming, it has not been used in previous large-scale studies. Automatic visceral fat calculation software would be a useful tool for large-scale research with accumulated CT volume data.

Although software for calculating the visceral fat volume from CT volume data has been developed [8, 9], the calculation range did not include the entire visceral fat volume. To date, there has been no published literature on automatic visceral calculation software that calculates the entire visceral fat volume, which would be indispensable for processing a massive amount of CT volume data sets.

Although some software for measuring the 3D fat volume from thick-slice 3D MR data has been developed [10, 11], MRI data is not accumulated over a wide abdominal range in medical institutions because MRI scanning with a wide abdominal range is not common.

The purpose of this study is to develop automatic calculation software to calculate the entire visceral fat volume from CT volume data and to evaluate its feasibility.

## 2. Materials and Methods

We used 24 sets of whole-body CT volume data with anthropometric measurement data. All the CT volumes (voxel size: 0.98 × 0.98 × 1.25 mm^3^) were scanned by a GE Light Speed CT scanner (16 detector row system, 120 kVp, auto (30–210) mA, 500 mm FOV, rotation time of 0.5 seconds, and moving table speed of 70 mm/second). These CT volumes were obtained from November 2006 to November 2008 at our institution. To represent various body types (from thin to obese) and both genders in equal numbers, three subjects were selected from each of four BMI categories for both sexes: values under 20, from 20 to 25, from 25 to 30, and over 30. This study was approved by the Ethical Review Board of our institution, and informed consent was obtained for the use of the CT volume data and anthropometric measurement data.

The true visceral fat region was defined as the region with CT values from −190 to −30 Hounsfield units (HU) [12] in the region between the diaphragm at the cranial end and the axial plane with the cranial edge of the pubic symphysis at the caudal end and in the peritoneal or retroperitoneal region manually segmented by a radiologist with 18 years of CT-reading experience. In the manual segmentation, the radiologist was supervised by another radiologist with experience in research on CT segmentation. The true visceral fat volume was measured from the true visceral fat region.

Software to automatically calculate the true visceral fat volume was developed using region segmentation based on morphological analysis with CT value thresholding. Details of the calculation method and implementation are described later. The calculated visceral fat volume is defined as the volume calculated automatically with this software.

The true visceral fat volume and calculated visceral fat volume of 24 subjects were compared by correlation analysis, and the ratio of error including calculated visceral fat volume was also evaluated.

Three major indices used as substitutes for visceral fat volumes, that is, the WC at the umbilicus level, the BMI, and the visceral fat area on an axial CT slice at the umbilicus level, were also evaluated by correlation analysis with the true visceral fat volume.

### 2.1. Development of Automatic Visceral Fat Volume Calculation Software

**Figure 1:**
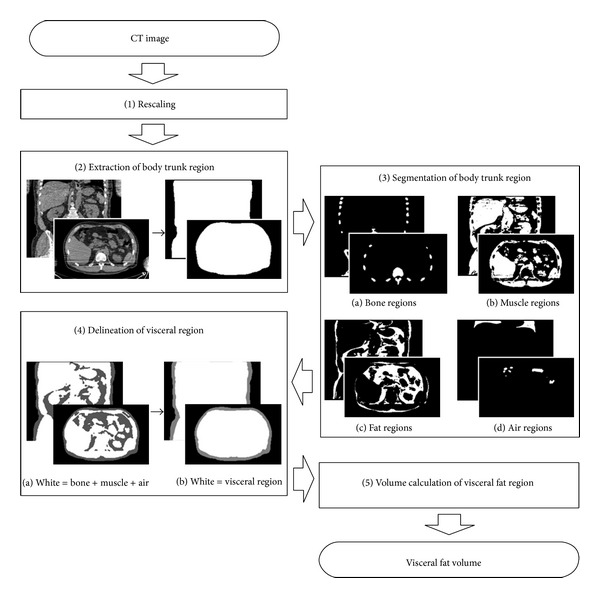
Flowchart of proposed fat volume calculation method.

Figure 1 shows a flowchart of the fat volume calculation method. Details of the method implemented for automatic visceral fat volume calculation method are given in the following.

**Step 1 (Rescaling)**

To reduce the calculation time for image processing, the CT volume data is scaled to half the original size by changing the voxel size to 1.96 × 1.96 × 2.5 mm^3^.

**Step 2 (Extraction of the body trunk region)**

The body trunk region is extracted by threshold processing and connection component analysis in each axial slice. In each slice, the largest binarized area with a CT value of −400 HU or higher is selected (corresponding to a region of bone, muscle, and fat). Moreover, the areas with a CT value of lower than −400 HU that do not touch the edge of the slice are also selected (corresponding to lung and intestinal gas regions). The selected regions are connected in three dimensions, and morphological closing is performed using a spherical kernel with a radius of 1.5 voxels.

**Step 3 (Segmentation of the body trunk region)**

The body trunk region is segmented into bone, muscle, fat, and air regions by performing various processes on the 3D volume as follows.

*Bone Region. *Voxels with a CT value of 200 HU or higher are selected in the body trunk region. The selected group of voxels is dilated using a spherical kernel with a radius of 1.5 voxels. A connected voxel component with the largest volume is selected as a seed region of bone. Since the seed region does not include low-HU regions such as medullary cavities, blank filling is performed in the seed region. The blank bone regions, which are negatives of the seed region and do not touch the edge of the CT volume, are selected. To sum the seed region and the blank regions, morphological erosion is applied with a spherical kernel of 1.5 voxel radius. 

*Muscle Region*. The connected voxel components that satisfy the following conditions are selected: (1) a CT value of 30 HU or higher, (2) no bone contained in the component, (3) a distance from the body surface of five voxels or more, and (4) a volume of 20 voxels (≒190 mm^3^) or more. 

*Air Region*. Connected voxel components with a CT value of less than −220 HU are selected. 

*Visceral and Subcutaneous Fat Region*. Connected voxel components whose CT value ranges from −190 to −30 HU [12] are selected from the body trunk region, excluding the combined regions of bone, muscle, and air. Morphological closing with a spherical kernel of 1.0 voxel radius is applied to the combined regions beforehand to avoid the misextraction of false positive regions from intermuscular fat regions, the neighborhood of the air region boundary having similar CT values to the fat regions, and image noise.

Next, the superior-inferior range of fat volume measurement is defined as follows. The superior end surface is obtained by radial basis function interpolation [13] for the caudal planes of the left and right lung regions, which are the largest and the second largest air regions. The inferior limit is defined as the horizontal plane including the pubic symphysis. To detect the level of the pubic symphysis, the anteriormost bony voxel is searched in a midsagittal slab whose thickness is 9.8 mm (=5 voxels).

**Step 4 (Delineation of the visceral region)**

From the bone, muscle, and air regions obtained by the above processing, the visceral region is drawn to include the abdominal wall muscle, abdominal organs, and visceral fat. Namely, morphological dilation processing is performed using a spherical kernel with a radius of 20.0 voxels on the seeds of the visceral region consisting of muscle, bone, and air regions. After the blanks of the dilated combined region are extracted and added to the dilated region, morphological erosion using a spherical kernel with a 20.0 voxel radius is applied to the combined region.

**Step 5 (Volume calculation of the visceral fat region)**

All fat regions are classified as either visceral fat tissue or subcutaneous fat tissue. Fat regions inside the visceral region are classified as visceral fat regions, while fat regions outside the visceral region are classified as subcutaneous fat regions.

In this study, the fat volume calculation software based on the proposed method is implemented in C++ language. The developed software handles DICOM-format CT data. The developed software is operated through CIRCUS CS (Clinical Infrastructure for Radiologic Computation of United Solutions Clinical Server) [14], which is a web-based integrated platform system for the development and assessment of various types of medical image analysis software.

The computer used to calculate the visceral fat volumes has an Intel Quad Xeon CPU 2.0 GHz processor and 3.0 GB RAM with Windows XP SP 2 installed.

## 3. Results

**Figure 2:**
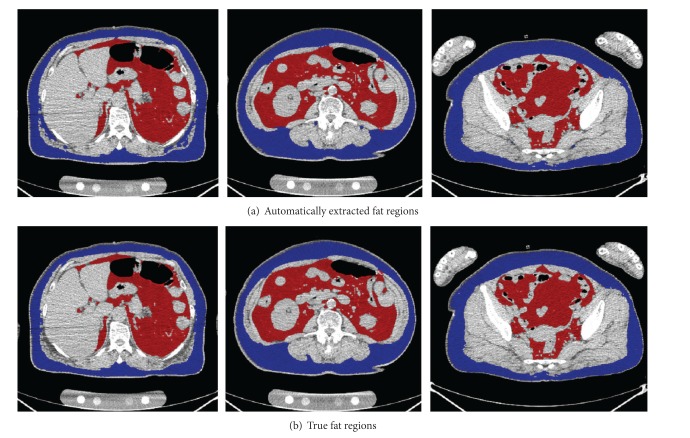
Example of successful automatic extraction of fat regions. Red/blue regions are visceral/subcutaneous fat regions.

Automatic calculation results for the visceral fat volume from all 24 sets of CT volume data were successfully obtained with the developed software. The average calculation time was 252.7 seconds with a standard deviation of 66.7 seconds. A selection of automatically segmented fat regions is shown in Figure 2.

**Figure 3:**
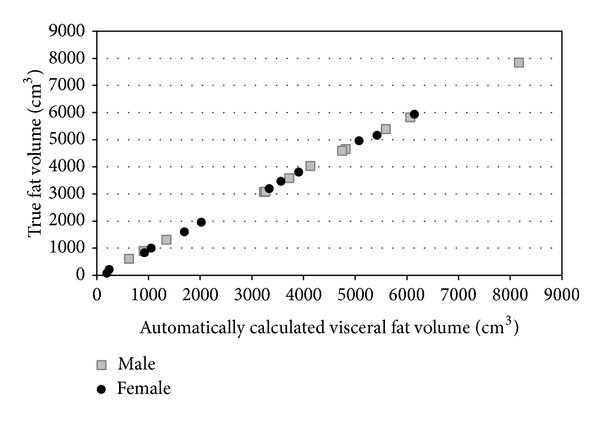
Correlation between automatically calculated visceral fat volumes and true volumes. The correlation coefficient for the female data set is 0.9998 and the coefficient for the male data set is 0.9999.

The true visceral fat volume and calculated visceral fat volume for each data set are shown in Table 1. Scattered correlation diagrams between the true visceral fat volume and the visceral fat volume calculated with our software showed a strong linear correlation (Figure 3). The correlation coefficients between the true visceral fat volume and the automatically calculated visceral fat volume were 0.9998 for males and 0.9995 for females. The median error ratio on the automatic calculation was 3.73% for males and 4.76% for females. Higher error rates were seen for cases with a low BMI.

**Figure 4:**
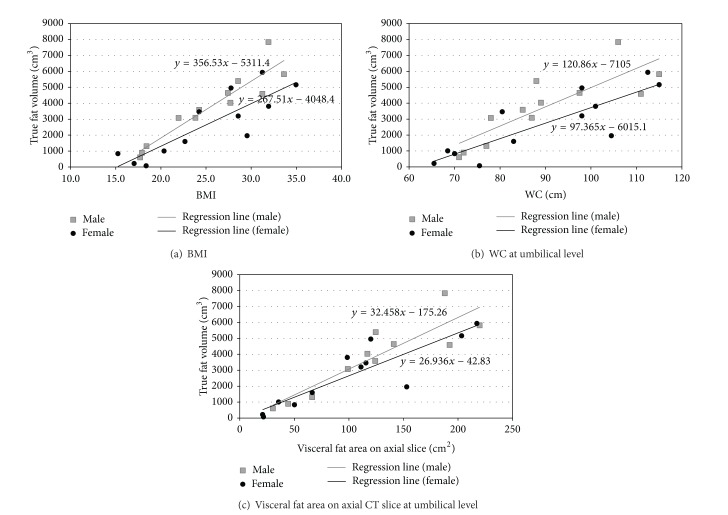
Linear correlations between the true visceral fat volume and the substitute indices for the visceral fat volume and their linear regression lines.

All these substitute indices (BMI, WC at umbilicus level, and area of 2D visceral fat region on axial CT slice at umbilicus level) showed a positive correlation with the true visceral fat volume (Figure 4) and also with the calculated true visceral fat volume. The correlation coefficients of the three substitute indices are shown in Table 2. The correlation coefficients differed with the genders and the substitute index.

## 4. Discussion

Software to automatically calculate the entire visceral fat volume from CT volume data was successfully developed. The software proved to be feasible for calculating the visceral fat volume with high accuracy in a reasonably short time. To the best of our knowledge, this is the first software to automatically calculate the entire visceral fat volume in the region from the upper abdomen to the pelvis. By applying the developed software through the CIRCUS platform, a large quantity of CT volume data can be processed automatically. This will be a useful tool in large-scale research involving the visceral fat volume when accumulated CT volume data is available.

The correlations with the substitute indices showed that they are not perfect for predicting the actual visceral fat volume. However, since these results were obtained using a small number of CT volume data sets, larger data sets are necessary to perform an accurate evaluation of the correlations between the indices and the fat volume.

**Figure 5:**
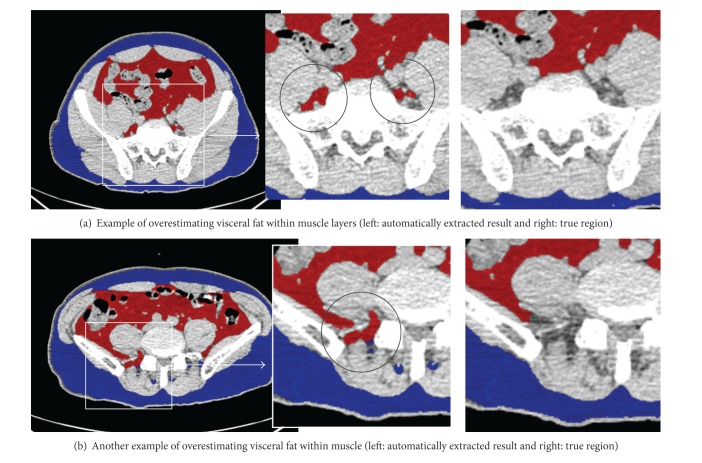
Examples of overestimation of visceral fat area by the proposed software.

In the automatic extraction of visceral fat regions, small false positive regions were often observed in the muscle gap (shown in Figure 5). To reduce these erroneous extractions, it is necessary to improve the extraction method for the abdominal region. If the abdominal region consisting of only the abdominal cavity region and the pelvic cavity region could be extracted, it would be possible to reduce the number of false positive errors.

In conclusion, we developed automatic calculation software to calculate the entire visceral fat volume from whole-body CT volume data. The developed software proved to be feasible for accurately calculating the visceral fat volume in a reasonably short time.

## Figures and Tables

**Table 1:** List of data sets with substitute indices for visceral fat volume, calculated true visceral fat volume, and automatically calculated visceral fat volume.

	Height (m)	Weight (kg)	Substitute indices			Visceral fat volumes		Absolute error of visceral fat volume (cm^3^)
			BMI (kg/m^2^)	Waist circumference (cm)	Visceral fat area (cm^2^)	True (cm^3^)	Calculated (cm^3^)	
Female	1.64	41	15.2	70.0	50	833	926	93
	1.57	42	17.0	65.5	21	216	243	27
	1.53	43	18.4	75.5	22	73	195	122
	1.47	44	20.4	68.5	36	1003	1054	51
	1.44	47	22.7	83.0	66	1598	1697	99
	1.56	59	24.2	80.5	116	3463	3565	102
	1.53	65	27.8	98.0	120	4955	5076	121
	1.40	56	28.6	98.0	111	3198	3340	142
	1.55	71	29.6	104.5	153	1955	2026	71
	1.57	77	31.2	112.5	217	5936	6149	213
	1.47	69	31.9	101.0	99	3805	3910	105
	1.55	84	35.0	115.0	203	5159	5426	267

Male	1.68	50	17.7	71.0	30	603	624	21
	1.62	47	17.9	72.0	44	891	908	17
	1.79	59	18.4	77.0	66	1306	1348	42
	1.68	62	22.0	78.0	99	3074	3239	165
	1.56	58	23.8	87.0	99	3081	3266	185
	1.65	66	24.2	85.0	124	3583	3726	143
	1.75	84	27.4	97.5	141	4647	4820	173
	1.71	81	27.7	89.0	117	4029	4137	108
	1.61	74	28.5	88.0	125	5393	5595	202
	1.58	78	31.2	111.0	192	4587	4748	161
	1.77	100	31.9	106.0	188	7837	8170	333
	1.80	109	33.6	115.0	220	5819	6069	250

**Table 2:** Correlation coefficients with the substitute indices for visceral fat volume and true/calculated visceral fat volume.

		Visceral fat volume	
		True	Calculated
Female	BMI	0.8540	0.8550
	Waist Circumference	0.8473	0.8510
	Visceral fat area	0.8826	0.8860

Male	BMI	0.9321	0.9304
	Waist Circumference	0.8378	0.8369
	Visceral fat area	0.8904	0.8901
